# Severe Post-COVID-19 dysautonomia: a case report

**DOI:** 10.1186/s12879-022-07181-0

**Published:** 2022-03-03

**Authors:** Joan Bosco, Ruwanthi Titano

**Affiliations:** 1 Division of General Internal Medicine, Department of Medicine, Icahn School of Medicine at Mount Sinai, Mount Sinai, Beth, Israel; 2 Division of Cardiology, Mount Sinai, Beth, Israel

**Keywords:** COVID-19, Post-acute sequalae of COVID-19 (PASC), Dysautonomia, Postural orthostatic tachycardia syndrome (POTS), Case report

## Abstract

**Background:**

The emergence of dysautonomia as a consequence of severe acute respiratory syndrome coronavirus 2 (SARS-CoV-2; or COVID-19) is becoming more prevalent. We have seen evidence in several post-COVID patients and in the literature of varying degrees of autonomic dysfunction. Symptoms, among others, include inappropriate tachycardia, sweating, anxiety, insomnia and blood pressure variability from the effects of excessive catecholamine, as well as cognitive impairment, fatigue, headaches and orthostatic intolerance from decreased brain perfusion.

**Case presentation:**

We present a case of severe dysautonomia in a previously healthy 27-year-old runner. About five weeks after her initial mild COVID-19 infection, the patient began to develop weakness, which progressed into severe post-exertional fatigue, slowed cognition, headaches, blurred vision and generalized body aches. She also endorsed palpitations, especially when getting up from a seated or lying position as well as with mild exertion. She became reliant on her husband for help with her activities of daily living. Exam was significant for orthostasis; laboratory workup unremarkable. Over the following months, the patient’s symptoms have improved slowly with fluid and sodium intake, compression stockings and participating in a graduated exercise program.

**Conclusions:**

Dysautonomia as a consequence of infection with COVID-19 is becoming increasingly discussed, especially as more patients recover from COVID-19. This is a case of a non-hospitalized patient with a mild initial presentation and significant, debilitating dysautonomia symptoms. More research on its pathophysiology, especially in relation to a precedent viral insult, as well as its treatment, is needed.

## Background

The emergence of dysautonomia as a consequence of severe acute respiratory syndrome coronavirus 2 (SARS-CoV-2; or COVID-19) is becoming more prevalent, from published case reports [1, 2] to its acknowledgement in retrospective studies characterizing both acute and delayed COVID-19 neurologic symptoms [3, 4]. Considered to be an improper functioning of the sympathetic or parasympathetic nervous systems, dysautonomia can present in many ways, including “labile blood pressure, orthostatic hypotension, impotence, bladder dysfunction and alterations in bowel functions” [1]. When dysautonomia manifests in the form of postural orthostatic tachycardia syndrome (POTS), patients report dizziness, lightheadedness, fatigue and tachycardia when standing from a sitting or lying position. Dysautonomia has been associated with several non-infectious conditions, from diabetes mellitus to Parkinson’s disease, as well as with viral infections, including, among others, HIV, hepatitis C, mumps, and Epstein-Barr virus [1].

The association of dysautonomia, particularly in the form of POTS, with chronic fatigue syndrome and/or myalgic encephalomyelitis (CFS; ME) is also becoming more understood. In fact, one of the 2015 Institute of Medicine’s diagnostic criteria for CFS/ME includes orthostatic intolerance, or “worsening of symptoms upon assuming and maintaining upright posture” [5]. CFS/ME has been associated with several viruses, including the 2003 severe acute respiratory syndrome coronavirus (SARS-CoV; 6), and has been recently garnering media attention as a post-acute consequence of SARS-CoV-2 infection. Below, we describe a dramatic case of POTS in a COVID-19 patient.

## Case presentation

A 27-year-old previously healthy female runner presented as an outpatient with lingering symptoms six months after her initial COVID-19 infection. Her initial symptoms lasted about two weeks and were mild; she was not hospitalized and did not receive any medical interventions. She had a positive COVID-19 polymerase chain reaction (PCR) by nasal swab five days into her illness.

About five weeks after the start of her initial symptoms, she visited the emergency department (ED) due to two weeks of progressive generalized weakness affecting her ability to move her extremities and ambulate. Work-up at this time was negative, including influenza swab, pregnancy test, urinalysis, complete blood count, comprehensive metabolic panel, and chest x-ray. Head imaging was not performed. She regained mobility and strength over the next three days.

The patient felt well enough to attempt to return to work about a month later, but only lasted a few days before she began to experience fatigue and flu-like symptoms. She went to an outpatient clinic where she again had a largely unremarkable lab workup, including complete blood count, comprehensive metabolic panel, thyroid function tests, and Lyme antibodies. Notably, at this time she was found to have a positive Epstein Barr Virus Viral Capsid Antigen (EBV-VCA) IgG antibody (416.00 U/mL; positive is > 21.99 U/mL); an equivocal EBV-VCA IgM antibody (36.70 U/mL; equivocal is 36-43.99 U/mL) and a negative EBV Nuclear Antigen IgG antibody.

Symptoms continued to progress over the next two months, including worsening post-exertional fatigue, slowed cognition with increased forgetfulness and difficulty concentrating, headaches, blurred vision and generalized body aches and weakness. She also endorsed palpitations, especially when getting up from a seated or lying position as well as with mild exertion. She noted frequent “muscle spasms and twitches” and burning in her feet at night. She became reliant on her husband for most of her Instrumental Activities of Daily Living (IADLs), and some of her Activities of Daily Living (ADLs) including grooming and bathing; she had to be carried up and down stairs.

About 5 months after her initial symptoms, the patient returned to the emergency department after attempting an exercise program, after which she developed “uncontrollable shaking,” diarrhea and extreme exhaustion. She again had an unremarkable workup. The patient presented to us as an outpatient about two weeks after. She endorsed worsening of the aforementioned symptoms and was now in a wheelchair. Medications at the time of her visit included oral contraceptives, paroxetine and medical marijuana (the latter two were initiated since her COVID-19 infection).

**Fig. 1:**
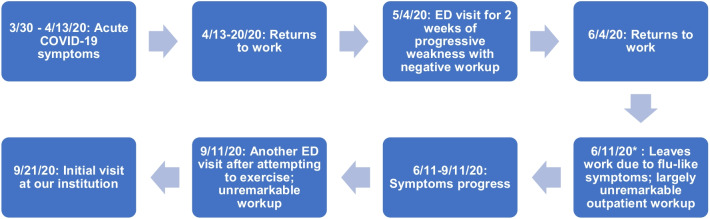
A graphic timeline of patient’s symptoms

*Date is approximate.

Exam was remarkable for an increase in heart rate of greater than 30 beats per minute (bpm) upon rising from a lying position (vital signs while lying down: blood pressure 112/70, heart rate 60–65 bpm; vital signs upon standing: blood pressure 112/70; heart rate 91 bpm). Patient was alert, oriented and conversant, albeit with several instances of repeating what she had previously said. Her neurologic exam was within normal limits, including normal pupillary light reflex (direct and consensual response). Figure 1.

Initial workup done at our office visit included normal complete blood count, comprehensive metabolic panel, estimated sedimentation rate, C-reactive protein, urinalysis, thyroid function panel, Vitamin B12 and Vitamin D levels, serum protein electrophoresis and immunofixation panel, rapid plasma reagin, iron and ferritin levels, hemoglobin A1C, beta-2-glycoprotein antibodies, cardiolipin antibodies and electrocardiogram. COVID-19 antibody titer was robustly positive.

One week later the patient saw cardiology, with whom she had a 10-minute active stand test in the office as an initial screening for POTS. This was positive for a greater than 30 bpm increase in heart rate within the first two minutes of standing. Throughout the duration of the test the patient endorsed shakiness, headache and subjective temperature change in her extremities. There was also rapid recovery to baseline resting heart rate within one minute of lying down in a supine position after upright testing. These findings are indicative of POTS.

Additional cardiac workup included a normal transthoracic echocardiogram and a dobutamine stress echocardiogram that was negative for ischemia and angina, but with an exaggerated heart rate response to exercise and below average functional capacity. The patient also underwent fludeoxyglucose (FDG) F-18 PET/MRI cardiac imaging which showed diffuse low grade FDG uptake throughout the myocardium consistent with low level physiologic uptake, and physiologic, nonspecific gadolinium uptake at the right ventricular insertion points on delayed enhancement gadolinium imaging. These findings are not indicative of active inflammation or fibrosis such as with acute or subacute myocarditis or residual scarring.

About two months after her initial presentation to our office, the patient started a post-COVID rehabilitation and physical therapy program. Over the next six months, she graduated from recumbent to seated and then standing/walking exercises. She implemented lifestyle changes, including increasing her fluid and sodium intake and wearing compression stockings. A year out from her initial infection, she is once again independent in her activities of daily living, although she is still not able to return to work.

## Discussion and conclusions

We present a case of severe dysautonomia in a previously healthy young patient. In our practice, this was the index case of a non-hospitalized patient with a mild initial COVID-19 presentation and significant, debilitating dysautonomia symptoms. We hope that this report will add to the ever-growing body of literature on Post-Acute Sequelae of COVID-19 infection (PASC) that may be overlooked or mistaken for another etiology. Although this case is a dramatic presentation, we have seen evidence of dysautonomia in several other post-COVID patients, with varying degrees of severity and disability. Symptoms, among others, include inappropriate tachycardia, sweating, anxiety, insomnia and blood pressure variability from the effects of excessive catecholamine, as well as cognitive impairment, post-exertional fatigue, headaches and orthostatic intolerance from decreased brain perfusion [4, 7, 8].

In severe cases, medications such as beta blockers, ivabradine, fludrocortisone or midodrine can be used for symptomatic management of heart rate and blood pressure dysregulation. The majority of patients, including the patient in this case, will improve with lifestyle changes such as adequate fluid and sodium intake, changing positions slowly, wearing compression stockings, and participating in graduated exercise programs to retrain the autonomic nervous system and correct cardiac deconditioning.

We would like to acknowledge the potential confounding variable of the patient’s positive EBV serology. It’s possible that the patient also had acute infectious mononucleosis (or an IM reactivation) during the same timeframe; the anti-VCA IgM could also have been a false positive. However, the patient’s symptoms are consistent with other post-COVID patients we have treated as well as seen in the literature [7, 8]. We do not suspect that her symptoms can be attributed solely to acute or reactivated IM infection.

In conclusion, there is growing awareness of dysautonomia as a subacute and chronic consequence of infection with COVID-19. More research on its pathophysiology, especially in relation to a precedent viral insult, is needed. Additionally, more research is needed to determine susceptibility to developing dysautonomia as well as treatment tailored specifically to post-COVID patients. Thus far, we have seen that recovery can be a slow, gradual process, but, over time, significant improvement does seem to be possible.

## Data Availability

All data generated or analyzed during this study are included in this published article. Specific laboratory or imaging data are available from the corresponding author on reasonable request.

## Abbreviations

ADLs: Activities of Daily Living
BPM: Beats per Minute
COVID-19: Coronavirus Disease 2019
CFS: Chronic Fatigue Syndrome
EBV: Epstein-Barr Virus
EBV-VCA: Epstein Barr Virus Viral Capsid Antigen
ED: Emergency Department
FDG: Fludeoxyglucose
HIV: Human Immunodeficiency Virus
IADLs: Instrumental Activities of Daily Living
IgG: Immunoglobulin G
IgM: Immunoglobulin M
ME: Myalgic Encephalomyelitis
MRI: Magnetic Resonance Imaging
PASC: Post-Acute Sequelae of COVID-19 infection
PCR: Polymerase Chain Reaction
PET: Position Emission Tomography
POTS: Postural Orthostatic Tachycardia Syndrome
SARS-CoV: Severe Acute Respiratory Syndrome Coronavirus
SARS-CoV-2: Severe Acute Respiratory Syndrome Coronavirus 2
